# IgA Nephropathy Mimicking Lupus Nephritis in Autosomal Dominant Polycystic Kidney Disease with Positive ANA and Anti-dsDNA: A Diagnostic Pitfall

**DOI:** 10.51894/001c.165695

**Published:** 2026-07-31

**Authors:** Sai Medavaka, Joshua Marwede, Manisha Garg

**Affiliations:** 1 Henry Ford - Providence

## 101

### Background

The coexistence of IgA nephropathy (IgAN) and autosomal dominant polycystic kidney disease (ADPKD) is exceedingly rare and creates significant diagnostic challenges. Clinical features of IgAN may overlap with other glomerular diseases, including lupus nephritis, particularly when autoimmune serologies are positive. Differentiating these conditions is critical because treatment implications are markedly different. We report a rare case of biopsy-proven IgAN in a patient with ADPKD whose presentation, accompanied by positive ANA and anti–dsDNA antibodies despite no prior history of systemic lupus erythematosus (SLE), initially suggested lupus nephritis.

### Case Presentation

A 57-year-old woman with known ADPKD presented with acute kidney injury, hematuria, and heavy proteinuria. Laboratory evaluation revealed positive antinuclear antibodies (ANA) and anti–double stranded DNA (dsDNA) antibodies, raising concern for possible lupus nephritis despite no prior history of SLE. Given the diagnostic uncertainty, a renal biopsy was performed. Histopathology demonstrated necrotizing and crescentic glomerulonephritis with IgA-dominant immune complex deposition and C3-dominant staining. Immunofluorescence lacked the “full-house” pattern typical of lupus nephritis, and no subendothelial wire-loop or membranous features were present. These findings confirmed IgA nephropathy and effectively excluded lupus nephritis. The patient was treated with high-dose corticosteroids and supportive renal care with close nephrology follow-up.

### Discussion

Positive autoimmune serologies alone do not establish a diagnosis of lupus nephritis. ANA and dsDNA antibodies may be detected in chronic kidney disease, infections, or other inflammatory states. IgA nephropathy may clinically mimic lupus nephritis due to shared features such as fatigue, hematuria, proteinuria, and acute kidney injury. In this setting, the immunofluorescence pattern on biopsy is essential for diagnosis. ADPKD further complicates evaluation because baseline chronic kidney disease and proteinuria may obscure superimposed glomerular pathology, and clinicians may hesitate to perform renal biopsy due to cystic kidneys. Only a limited number of cases describing ANA- and dsDNA-positive IgAN have been reported, and even fewer involve concurrent ADPKD.

### Conclusion

This case highlights the diagnostic complexity of glomerular disease in patients with ADPKD and emphasizes that positive serologies should not replace histopathologic confirmation. Renal biopsy remains the gold standard for diagnosis and is essential to avoid misclassification and inappropriate immunosuppressive therapy.

